# Memristive crypto primitive for building highly secure physical unclonable functions

**DOI:** 10.1038/srep12785

**Published:** 2015-08-04

**Authors:** Yansong Gao, Damith C. Ranasinghe, Said F. Al-Sarawi, Omid Kavehei, Derek Abbott

**Affiliations:** 1 School of Electrical and Electronic Engineering, The University of Adelaide, SA 5005, Australia; 2 Auto-ID Labs, School of Comupter Science, The University of Adelaide, SA 5005, Australia; 3 Centre for Biomedical Engineering, School of Electrical and Electronic Engineering, The University of Adelaide, SA 5005, Australia; 4 Functional Materials and Microsystems Research Group, School of Electrical and Computer Engineering, Royal Melbourne Institute of Technology, Victoria 3001, Australia

## Abstract

Physical unclonable functions (PUFs) exploit the intrinsic complexity and irreproducibility of physical systems to generate secret information. The advantage is that PUFs have the potential to provide fundamentally higher security than traditional cryptographic methods by preventing the cloning of devices and the extraction of secret keys. Most PUF designs focus on exploiting process variations in Complementary Metal Oxide Semiconductor (CMOS) technology. In recent years, progress in nanoelectronic devices such as memristors has demonstrated the prevalence of process variations in scaling electronics down to the nano region. In this paper, we exploit the extremely large information density available in nanocrossbar architectures and the significant resistance variations of memristors to develop an on-chip memristive device based strong PUF (mrSPUF). Our novel architecture demonstrates desirable characteristics of PUFs, including uniqueness, reliability, and large number of challenge-response pairs (CRPs) and desirable characteristics of strong PUFs. More significantly, in contrast to most existing PUFs, our PUF can act as a reconfigurable PUF (rPUF) without additional hardware and is of benefit to applications needing revocation or update of secure key information.

The earliest known lock, resembling the mechanical locks of this century, is estimated to be 4,000 years old—a large wooden lock and key from the palace of Khorasabad in Niveveh. Modern security systems still keep valuables under *lock and key* in order to ensure the safety and authenticity of goods, information or identities. Locks now, however, can refer to electronic security systems with keys that are coded in, for example, magnetic strips or silicon chips.

Since the invention of locks and keys—physical, electronic or combined—the essence of keeping a good security system effective is protecting the key. This is one of the reasons we keep the physical key to our offices on a personal key chain, our electronic access cards on lanyards, and never reveal our computer passwords. Whilst physical locks can be picked, in the digital and electronic worlds hackers have developed both invasive and non-invasive physical tampering methods, such as micro-probing, laser cutting, and power analysis^1^, in order to extract digitised secret information from the integrated circuits (ICs) imprinted on devices such as credit cards. Even tamper proofing techniques used in smartcards—such as cutting power or tripping tamper-sensitive circuitry that clears out the secret information—to protect secret keys have been shown to be vulnerable to physical attack^1^. Therefore, the problem is storing digital information in a device in such a way that is resistant to physical attacks.

Recently, the growing new area of PUFs is receiving increased attention because a PUF offers a solution by extracting secret key information from a complex physical system. Therefore, unlike conventional applications, more randomness is a desirable property for building PUFs. They are favourable because they are easy to build but practically impossible to duplicate. This latter strength comes from the use of a micro-structure that is bound to tiny variations in physical properties resulting from numerous random features that occur during manufacturing to generate secret keys. When a PUF is physically stimulated, i.e., challenged, a response is generated by a complex interaction of the challenge with many or all of the random micro-structure features. Therefore, the use of PUFs for security is, extremely appealing, since the simple process of fabrication equips them with a unique and unclonable features for extracting secret keys. Besides cryptographic key generation, PUFs can be used for more complicated cryptographic protocols such as oblivious transfer (OT), bit commitment (BC), key exchange (KE), device authentication and identification^2^,^3^,^4^,^5^,^6^,^7^,^46^.

Conventional PUFs such as Ring Oscillator PUF (ROPUF)^2^, Arbiter PUF (APUF)^9^,^1^0, SRAM (Static Random Access Memory) PUF (SRAM PUF)^11^,^1^2 exploit uncontrollable process variations in conventional CMOS fabrication technology—a comprehensive overview of PUFs can be found in^13^,^1^4. Although technological developments in CMOS devices such as the FinFET extend the life of microelectronics, such developments are still expected to confront physical limitations imposed by the continuing trend towards smaller feature sizes. Consequently, CMOS based PUF designs will also face a roadblock in terms of providing secure physical unclonable functions in the future^15^. Furthermore, contemporary PUF designs also face challenges, such as exhaustive CRP access attacks to PUFs that possess a limited number of CRPs, model building attacks^16^,^47^, reliability deterioration due to device aging or extreme environment conditions. Strong PUFs^14^,^1^6 address the first two issues because they are capable of producing huge number of CRPs and are resistant to model building attacks. Reconfigurable rPUFs^17^ overcome the last two issues because they endow appealing features such as key renewal and a revocation ability by updating itself as a new PUF instance. Here, for the first time, we exploit the unique properties of nanoelectronics rather than CMOS technology to provide an opportunity for building a PUF design possessing integrated strong PUF and rPUF characteristics in one single PUF architecture. More significantly we are able to achieve reconfigurability that is impossible to reverse.

The nanocrossbar is in principle the simplest functional electrical circuit holding great promise in nanoelectronics due to its attractive, regular structure and simple implementation. The nanocrossbar array (Fig. 1a) consists of parallel horizontal wires on top and perpendicular vertical wires at the bottom. Nanocrossbars are usually combined with a passive two-terminal resistive device such as a memristor (in the literature, the terms *memristor* and *memristive device* are used interchangeably)^18^,^19^,^20^ shown in Fig. 1b at the cross-point aimed for data storage, computing, and neuromorphic applications. Together with nanocrossbar structures the unique properties of memristors such as non-volatility, switching behaviour and nanoscale dimensions present new opportunities for realizing ultra high density memory arrays^21^.

It has been shown that the memristor can be used to store digital states by utilizing its two distinct resistance values Fig. 1c,d namely the ON resistance and OFF resistance, referred to as *R*_ON_ and *R*_OFF_. However, memristors have inherent randomness at both the memristor device level—due to the cycle to cycle programming variations of the device—as well as the fabrication process level (such as thickness and cross-sectional area variations)^22^,^2^3. These resistances are random variables with log-normal distribution values^19^. In Figs 2 and 3, we illustrate the distribution of these resistances after an initial programming step of randomly selected binary values in a nanocrossbar array. Undesirable resistance variation will deteriorate the ability to distinguish *R*_ON_ and *R*_OFF_ states during reading the state of a memristor in a nanocrossbar array (Fig. 1e). However, this large resistance variation is very desirable for constructing a PUF architecture. In addition, the large information density in the nanocrossbar enables strong PUF characteristics. Moreover, the unique C2C (Cycle-to-Cycle) programming variation of memristance allows building PUF architectures with reconfigurable characteristics. Furthermore, our mrSPUF in this report realizes a potentially low cost security primitive because the memristor-based nanocrossbar is easy to fabricate and compatible with CMOS fabrication processes. All aforementioned advantages motivate the design of our mrSPUF.

The proposed mrSPUF architecture that combines nanocrossbar and current mirror controlled ring oscillators to realize a strong and reconfigurable PUF is unique and has not been considered in the past to the best of our knowledge. Our architecture allows the extraction of secret information by exploiting the abundant and readily available variations in nanodevices. We summarize our contributions as: i) A novel PUF architecture that exploits the fabrication variations inherent in nano-electronic devices. In particular we exploit the significant variations in the resistance values in a nanocrossbar structure based resistive memory to build mrSPUF; ii) We evaluate mrSPUF and demonstrate its superior performance with respect to fundamental performance metrics: uniformity; diffuseness; uniqueness; and reliability by conducting extensive studies; iii) We demonstrate the capability of the proposed structure in generating a significantly large number of CRPs and, consequentially, we show that mrSPUF is a candidate of strong PUF. vi) We demonstrate that mrSPUF can act as a rPUF relying on C2C programming variations of memristors and, which has never been exploited in memristor based PUFs, to the best of our knowledge. Moreover, the architecture integrates nanocrossbar array with current mirror-controlled ring oscillators is also unique.

## Results

### mrSPUF Architecture

Our proposed mrSPUF architecture aims to exploit the substantial variations in individual memristors that can be realized in high density with minimal area overhead using a nanocrossbar shown in Fig. 4. To capture variations of memristors and increase the number of CRPs, we employ multiple individual memristors to control the oscillation frequency of a CM-RO that translates analog variations to digital frequency counts (see Fig. 4d). To enable reconfigurability and generate responses to challenges, our mrSPUF architecture also includes key circuits to both program and re-program the nanocrossbar (see *programming control circuitry* shaded in Fig. 4), apply challenges using the decoder block and the analog MUXs block, and extract digital responses using the CM-RO, counter and comparator in Fig. 4.

**Figure 1:**
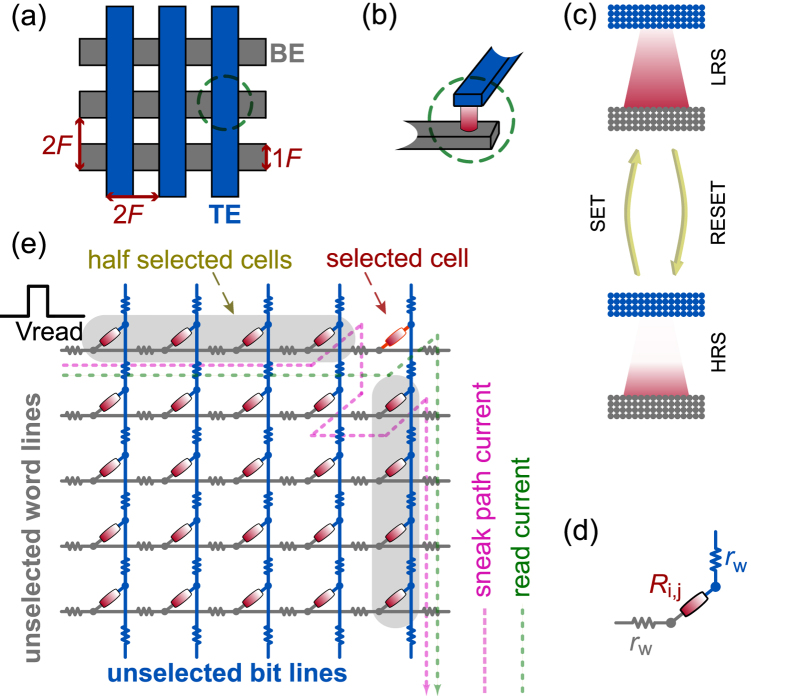
Nanocrossbar array and a memristive device. (**a**) Nanocrossbar array of memristive devices, where each memristive device is located at the crosspoint of a top and a bottom electrode as shown in (**b**). (**c**) Illustrates the operation principles of a memristive device. There is a concentration gradient of ions which can be moved back and forth using an applied electric field. The memristive device switches from HRS (High Resistance State) to LRS (Low Resistance State) with a positive potential difference between the bottom electrode and top electrode corresponding to SET switching as one or more conductive filaments grow or form, while it switches from LRS to HRS with a negative potential difference between the bottom electrode and top electrode corresponding to RESET switching as the filaments are disrupted. Once a memristive device has been programmed its memristance does not change even if its power supply is disconnected unless a voltage higher than threshold voltage is applied across the device. Note that this is a very simple illustration of the memristive switching and does not cover all device-physics aspects of the switching. (**d**) An interconnection model of one memristive device in the array where *r*_*w*_ is the segment nanowire resistance and *R*_*i*,*j*_ is the resistance of the memristive device at the crosspoint of the *i*-th bottom and *j*-th top electrode. (**e**) Reading scheme. When reading a targeted memristive device (selected cell), reading voltage is applied to the selected word line and the current passing through the selected bit line is sensed to determine the state of the memristive device. For other unselected word and bit lines, they can be connected to ground or to a high impedance (floating). During reading it is important to note that there also exists many sneak path currents (purple dashed line) besides the desired read current (green dashed line).

Although the nanocrossbar offers promising low area overhead circuitry for storing high-density information, the main problem it faces is the presence of sneak path currents^19^. A memristive device is read from the nanocrossbar by applying a voltage to the selected word line of the nanocrossbar array. This will result in a current that flows through the selected device. The current through the selected bit line is then sensed to read the analog/digital state of the memristor. In a practical implementation of such nanocrossbar architecture, in addition to the current through the targeted memristor, there exists a number of other current paths that are commonly referred to as *sneak path* currents that result in an inaccurate reading of the targeted memristor device value (see Fig. 1e). This effect is illustrated in Fig. 5. It should be noted that sneak path current in the nanocrossbar array mitigates the effect of process variations (reduces the standard deviation) in individual memristors during readout. A larger standard deviation is desirable for PUF that derives its security by exploiting process variation. To suppress sneak path currents and therefore maintain the readout resistance variations of memristors in crossbar, a number of techniques can be used^19^. Three of the leading techniques at the centre of attention in today’s industry and academic research community to suppress sneak path currents are: i) an intrinsic current-rectifying behaviour^19^,^2^4 that is translated into an extremely high current-voltage nonlinearity as shown in Fig. 6; ii) using a highly nonlinear series element with a transistor-like or a diode-like behaviour; and iii) using a complementary resistive switches (CRS) to replace a single memristor in nanocrossbar array^25^,^2^6. Presently, the first solution appears more promising than the other two approaches due to its ability to maintain competing memory features such as high density and the highly nonlinear self-rectifying effects in these solid-state devices. As for CRS, the read operation is destructive and multilevel capability of memristive devices cannot be used.

The mrSPUF operation comprises a *Programming Phase* and a *Response Generation Phase*.

### Programming phase

The resistance variation is more prevalent in the *R*_ON_ state than in the *R*_OFF_ state due to the thickness of memristors demonstrated in^23^. Furthermore, in^27^ it is demonstrated that resistance is resilient to temperature and telegraph noise in the *R*_ON_ state more than in the *R*_OFF_ state. For these reasons only the *R*_ON_ state is used to construct the mrSPUF architecture (i.e. we initially program the entire nanocrossbar to store the logic value ‘1’) to reduce susceptibility to both temperature fluctuations and telegraph noise and consequently increase the reliability of the PUF architecture.

The programming control circuitry is shown in Fig. 4b,c. In programming phase, we set *P* = 1 to disconnect rows and columns from the analog multiplexers block and decoder block. Programming a memristor into the *R*_ON_ is carried out by a SET operation (Fig. 1c) achieved by applying S = *V*_SET_, a voltage that is higher than a memristor’s positive threshold voltage (Fig. 6), to the selected row (word line) and applying R = 0 to the selected column (bit line). All other unselected rows and columns are floating to reduce power consumption.

### Response generation phase

Once all the memristors in the nanocrossbar array are programmed into ON state, we set *P* = 0 to connect rows with analog multiplexers block and columns with decoder block. And simultaneously, set rows and columns connected to programming control circuitry to floating (Fig. 4b,c). Then mrsPUF is configured to generate responses to given challenges.

The decoder block selects one column of the nanocrossbar array. The analog multiplexers block selects a set of 2 × *i* rows acting as bit lines. In our mrSPUF design, ten memristors (2 × *i*, *i* = 5) in the same column are selected. Each selected memristor is then used to control the current in a single current mirror and consequently to starve the current in each inverter in the ring oscillator, to achieve a *current starved ring oscillator* structure (Fig. 4a,d) refereed to current mirror controlled ring oscillator (CM-RO). Therefore, the oscillation frequency of a CM-RO is predominantly a function of current through the memristor that in turn is related to the resistance of the memristor. Consequently, the oscillation frequency of each oscillator is a function of the selected memristors and measured using a counter. Subsequently, a response bit is generated by comparing the outputs from the two counters.

In general mrSPUF has two CM-ROs, each RO has *i* inverters, 2 × *i* memristors are randomly selected. So the total CRPs (NT_CRP_) in this configuration can be estimated as:


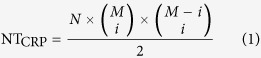


where *N* is the number of columns, *M* is the number of rows. Therefore, the number of inverters in CM-RO or the nanocrossbar array size or all of the design parameters can be altered according to the desirable number of CRPs.

The average frequency observed by using 5 inverters configured by 5 memristors is in the vicinity of 25 MHz. Therefore, an advantage of using the current starved ring oscillator is that the frequency is sufficiently low that an accurate counter is not required to measure the frequency of CM-RO.

### Fundamental PUF characteristics of mrSPUF

We evaluate the fundamental PUF characteristics of mrSPUF using uniformity, diffuseness, uniqueness, and reliability metrics. Detailed definitions of these metrics can be found in the Methods section. Generally, A PUF generates multiple bits responses. However, A single mrSPUF only produces a single response bit. To obtain multiple response bits, there are two solutions. First, one can duplicate mrSPUF architecture on the same chip with the same challenge as the input. Second, one can query a single mrSPUF with a set of challenges (multiple challenges) and concatenate the response. Here, we use the later approach for saving chip area.

#### Uniformity and diffuseness

Uniformity captures the proportion of ‘1’ and ‘0’ in responses of a PUF. Ideally, the proportion is 50% for truly random response. A PUF is expected to output different responses to different challenges and these responses can be treated as IDs of a PUF. Diffuseness is the degree of difference among different responses to different challenges for the same PUF. The expected diffuseness is 50%.

In order to evaluate uniformity and diffuseness we randomly apply 100 different challenge sets to one mrSPUF. Each challenge set has 128 challenges, subsequently, the 128 single response bits are concatenated to form a 128 multiple bits response. Each 128 bits response vector acts as an identifier (ID) of a given mrSPUF instance. The distribution of ‘1’ and ‘0’ among all response vectors is calculated representing uniformity. From Fig. 7a, it can be seen that both the probability of ‘1’ and ‘0’ are nearly 50%, which are 50.76% and 49.24% respectively. We calculate Hamming Distance (HD) between responses of these IDs to determine diffuseness. The diffuseness is 49.96% that is close to the ideal value of 50%, as shown in Fig. 7b.

#### Uniqueness

When the same challenge sets are applied to different PUF instances, the output responses are expected to be different. Uniqueness represents the ability to uniquely distinguish a particular PUF instance among a group of instances. Ideally, the HD between the responses to a same challenge from different PUF instances should be 50%.

We use 100 different mrSPUF instances to evaluate uniqueness and the result is shown in Fig. 8. The mean of HD of mrSPUF is 64.1 bits out of the 128 bits response and this value agrees with ideal value of 64 bits.

#### Reliability

Reliability measures the consistency with which a given PUF reproduces a response to repeated application of a given challenge. Ideally, a PUF reproduces the same responses corresponding to the same challenges for the same PUF. The reliability can also be assessed by Bit Error Rate (BER) measuring the percentage of flipped (error) bits in responses. Reliability and BER are complementary metrics. From a practical implementation point of view there is a need to evaluate reliability under different temperature and supply voltages.

We evaluate the reliability of 20 mrSPUFs. The results obtained are shown in Fig. 9. Response vector of 500 bits generated by using a set of 500 randomly selected challenges is collected by repeatedly testing each mrSPUF instances using four different supply voltages, namely, 0.9 V, 0.95 V, 1.05 V and 1.1 V. Here we use BER. The worst mean BER (Bit Error Rate) is 1.5% under −10% deviation from the nominal value of 1 V, while the circuit core temperature is kept at 27 °C.

The resistance temperature coefficient of a memristive device in ON state is similar to a metallic resistor^28^,^2^9. So we use temperature coefficients obtained from metallic resistors to conduct reliability evaluation under different core temperature settings. Temperature reliability tests were repeated for four different ambient temperatures (−20 °C, 0 °C, 50 °C, 85 °C) under a constant power supply voltage of 1.0 V. The worst mean BER observed is 4.9% when the temperature is 85 °C.

We also conducted a corner test in four combinations: (0.9 V, −20 °C); (0.95 V, −20 °C); (1.0 V, 27 °C); (1.05 V, 85 °C) and (1.1 V, 85 °C). The worst case occurs when the supply voltage is 0.9 V and temperature was reduced to −20 degree Celsius where the mean BER is 7.5%.

It is impractical to directly use the raw responses of mrSPUF as cryptographic keys. On one hand, output response to a given challenge slightly differ at times applied to the same PUF instance due to temperature and supply voltage fluctuations. However, cryptographic primitive requires that every bit of the key remains unchanged. On the other hand, cryptographic primitive such as RSA requires the key to meet specific mathematical properties unavailable from mrSPUF randomly responses^2^.

To generate cryptographic keys based on PUF responses that overcomes the aforementioned limitations, two steps should be followed. Firstly, ECC (Error Correction Code) like BCH code can be used to generate syndromes from stable PUF responses to subsequently correct erroneous bits in PUF responses. After correction, the corrected response can be directly used for cryptographic primitives such as AES (Advanced Encryption Standard) requiring no specific properties after simply hashing down to a desired length. Secondly, if specific mathematical properties are needed for cryptographic primitives, the corrected PUF output can be used as a seed to generate required cryptographic keys with the desired mathematical properties.

### Strong PUF characteristics of mrSPUF

Before delving into specifics, we first outline the characteristics expected from a strong PUF model^14^,^1^6:

- Impossible to build a duplicate or clone of a PUF instance physically,
- Generate a huge number of CRPs to prevent exhaustive measurement of responses to all possible challenges—full characterization of the PUF—within a limited time period, several days or weeks, demonstrated ideally by exponential CRPs in the number of challenge bits,
- Resilient to model building attacks when an adversary aims to build a complete model to predict responses to hitherto unseen challenges given a polynomial number of CRPs.

Similar to silicon PUFs the proposed mrSPUF meets the first feature since it exploits significant abundant and uncontrollable process variation in fabrication of nano devices, even manufacturer can not fabricate two identical mrSPUFs. The number of CRPs generated by a given nanocrossbar array size and the number of inverters stages in one CM-RO is calculated using Eq. (1) is described in Fig. 10. For example, using a nanocrossbar array with equal number of rows and columns (*N* = *M* = 100) and a 5-stage (*i* = 5) CM-RO, we can acquire 2.1811 × 10^17^ CRPs. Assuming a readout speed of 10^7^ bits/s for mrSPUF, it will take over 690 years for an attacker to fully readout all possible CRPs. In reality it will be greatly slower because CM-ROs average frequency is around 25 MHz and therefore it will take longer to accurately measure frequencies even if a high accuracy counter is used. Moreover, the starting period before RO frequency is stable will prolong the time for each measurement. Therefore our mrSPUF meets the second characteristic of strong PUFs. Furthermore, we also investigate model building attacks and demonstrate that mrSPUF is resistant to model building attacks (see section Additional Information). So the mrSPUF is a candidate strong PUF.

### Reconfigurability of mrSPUF

The concept of a reconfigurable PUF, first articulated in^30^, is the idea of imparting on to the same PUF realization the ability to change its behaviour to the same challenge. Instead of exhibiting static challenge-response behaviour, updatability of challenge-response behavior of reconfigurable PUF is desirable for some practical applications including the revocation or update of ‘secrets’ in PUF-based key storage and cryptographic primitives based on PUFs^31^. Since then a stronger definition of a reconfigurable PUFs has been proposed to ensure that the reconfiguration is difficult to reverse, even by an invasive attack measure^32^. Therefore the reconfiguration should not depend on a hidden device or parameter that can be influenced by an attacker, for example, part of the challenge or the location of the PUF structure in reconfigurable FPGA logic^33^.

Ideally, a rPUF is a PUF that can be updated using a methodology to alter the PUF into a new instance such that:

- The CRPs of rPUF are unpredictable after reconfiguration even if the CRPs of a rPUF before reconfiguration are known.
- The security properties of the rPUF are preserved after reconfiguration.
- Reconfiguration is uncontrollable and should not rely on updating a hidden parameter.

In^32^, authors present two rPUFs concepts. The first is a reconfigurable optical PUF, when this PUF is irradiated with a laser beam beyond normal operating conditions, the structure will change its internal configuration and subsequently refreshed CRPs can be obtained while the security properties are preserved. The second is PCM (Phase Change Memory) based rPUF. The PCM is a resistive device with a lower endurance characteristic because the switching (analog or digital) is a result of small area (≈1 nm) melt-down of device structure and switching between amorphous and crystalline material states. Although the authors in^32^ did not consider the significant problem of resistance drift in PCMs, the reconfigurability of the PCM-based PUF comes from the ‘long-lived random state that can be reconfigured at will’, precisely what is needed for an rPUF. A reconfigurable optical PUF is not practical for lightweight cryptographic primitives because its implementation especially the measurement part is expensive. In^17^ the authors further investigate the conceptual PCM-based rPUF^32^ and show its feasibility. However, the hash function and fuzzy extractor^45^ are always used since the uniqueness, reliability is unsatisfactory leading to a great overhead.

The internal change in ionic concentration gradient within nonionic memristors during switching (see Fig. 1c) results in different resistance values for *R*_ON_ and *R*_OFF_. This variation is referred to as C2C variation. Figure 11 illustrates the C2C variation in resistance values for 5000 reprogramming cycles. It is worth highlighting that components of this source of variation, as oppose to conventional CMOS, is a unique spatiotemporal source of variation. By utilizing this property, we can implement a reconfigurable PUF using mrSPUF because after reprogramming the memristors in the nanocrossbar, their resistances in *R*_ON_ will change randomly and therefore will meet the requirements for a rPUF.

#### Reconfigurable phase

Additional circuitry is not needed to enable the mrSPUF acts as an rPUF other than switching the memristor from ON state to OFF by applying R = |*V*_RESET_|, a voltage that is higher than absolute value of negative threshold voltage, to the selected column (bit line) and applying S = 0 to the selected row (word line). Then switching it back from OFF to ON state again by applying S = *V*_SET_ to the selected row and R = 0 to the selected column using the programming control circuitry shown in Fig. 4.

The advantage of using mrSPUF-based rPUF is low-cost overhead requiring no additional circuitry and unlimited configuration space—the number of times that rPUF can be evolved into a new PUF instance—as opposed to other rPUFs. More significantly, the reconfiguration is nearly impossible to reverse!

## Discussion

In contrast to ROPUF that uses an array of ROs, the proposed mrSPUF efficiently uses two *i*-stage CM-ROs which are re-configured using the nanocrossbar and consequently leads to a significant area reduction and ease of reading as the output frequency is substantially reduced to facilitate accurate counting. Also unlike the memristor-based PUF in^34^ where the goal is to sense the value of the resistance to determine the binary value (in *R*_ON_ or *R*_OFF_ state) of a target memristor in nanocrossbar array, we translate analog memristance value (here only *R*_ON_ state is used) into a frequency through CM-RO. The advantages of this implementation include: i) Use of significantly smaller number of ROs and only *i* inverter stages (in the demonstrated case, *i* = 5) to build each RO. ii) Generate a significantly improved number of CRPs. iii) Mitigate some of the undesirable variations in responses caused by power supply and temperature fluctuations as we employ a differential structure to generate a response bit. vi) Unlike the proposed structure in^34^ we do not need complex circuitry to readout a memory cell and we do not directly expose full physical information—binary value in memory—at each junction of a nanocrossbar array.

Besides our proposed memristor based PUF, there are some initial works addressed the feasibility of building up a memristor based PUF^34^,^35^,^36^,^37^. Both of these two studies^34^,^3^5 employ a time and voltage constrained write mechanism (weak-write) to force each memristor to an undefined logic region (neither logic ‘1’ or ‘0’). Subsequently, these memristors attain an unpredictable logic state due to process variations that influence memristance. Similar to SRAM PUF, a memristor PUF^34^,^3^5 is only capable of producing a limited number of CRPs. More significantly, the memristor based PUFs in^34^,^3^5 require a calibration procedure to determine the weak-write parameters (time and voltage) to force a memristor into undefined logic region before it can be used. The proposed PUFs exploit *static randomness* from process variations. However, it should be noted that the memristor has large C2C variations, which means reprogramming it using the same pulse period or voltage amplitude will cause unstable responses (dynamic randomness) for the same challenge with respect to the same PUF for the aforementioned memristor based PUFs. This C2C variations is not taken into account in the aforementioned memristor based PUF designs.

In^38^ the authors leveraged sneak path currents inherent in memristor-based nanocrossbars and bidirectional features to build up a nano PPUF (Public Physical Unclonable Function). Unlike PUFs, the security of the nano PPUF no longer relies on the secrecy of its physical parameters that define its uncontrollable variations and the model of a PPUF that exactly matches the PPUF hardware behaviour is publicly known to every one. The security of a PPUF is based on the time difference (several orders of magnitude) between fast execution time on PPUF hardware to acquire correct response and the much longer time required to compute the response correctly using the PPUF model. In fact, PUFs and PPUFs are hardware primitives with different requirements for authentication and other security services. Since nano PPUF always need accurate measurements of its physical model parameters to create the model of nano PPUF that is inconvenient and expensive. Although PPUFs provide an alternatives securely storing challenge response pairs, the high reliability requirement of PPUF designs still need to be addressed. We refer readers to^39^ for a more comprehensive overview.

Here we compare mrSPUF with other memristor based PUFs. However, comparison with the nano PPUF is not conducted because the nano PPUF has different application protocols and the most difference is that building a model of nano PPUF requires a very high accuracy measurement in provisioning phase.

In Table 1, whether nanocrossbar is used or not determines the circuit’s density. Since all the PUF performance data in Table 1 are from simulation, so the uniqueness and uniformity are all close to ideal value of 50%. We do not compare reliability and diffuseness performance because there is no such information in other PUFs listed in the table. *M* and *N* mean number of rows and columns in nanocrossbar array respectively for the proposed mrSPUF and the PUF structure in^34^. In this PUF structure^35^, *M* means the number of memristors used since there is no nanocrossbar used. In general, the number of CRPs is equal to the number of memristors in these two PUF designs^34^,^3^5. It can be seen our proposed mrSPUF can significantly expand the number of CRPs. Proposed memristor based mrSPUF distinguish from other memristor based PUFs in terms of reconfigurability and has the qualities of strong PUF. We propose the concept of exploiting the inherent C2C variations of memristor to build rPUF for the first time.

## Methods

### Simulation set-up

**Figure 3:**
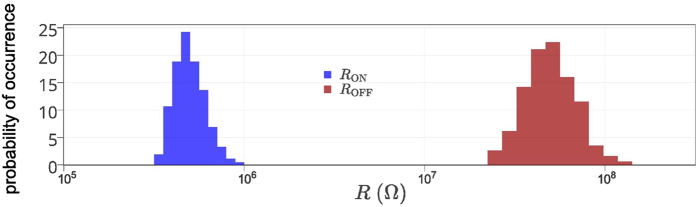
Histogram of resistance variation distribution. Experimental resistance variation distribution extracted from a 40 × 40 nanocrossbar array with 1600 memristors obtained from experimental data in^19^. Note that data for this graph was extracted by reading each crosspoint resistance (current sensing) in the crossbar, therefore, resistance of an individually isolated device may be higher.

**Figure 4:**
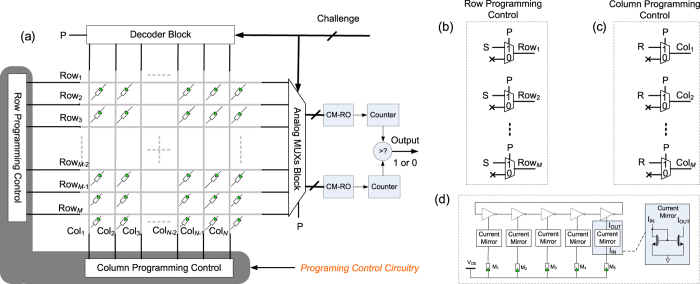
mrSPUF architecture. (**a**) Simplified mrSPUF architecture. All the memristors are in the ON state. The shaded *programming control circuit* comprises of the row programming control circuitry in (**b**) and column programming control circuitry in (**c**) which is employed to program memristors in nanocrossbar array before it acts as a PUF and facilitates reconfigurability of mrSPUF by subsequent reprogramming to refresh CRPs of mrSPUF to transform it into a new PUF instance. In contrast to the programing control circuit, the top decoder block and left analog multiplexers block, CM-ROs and counters enables the stimulation by a challenge and the extraction of a corresponding response. A challenge encoded as a vector of binary values (bits) is used to provide the address bits for both the analog multiplexers block and the decoder block. (**d**) CM-RO. Each current mirror starves only an inverter in the RO structure, where the bias memristor for each current mirror, *M*_*i*_, is selected from the nanocrossbar array. Although variation in the oscillation frequency of each RO is slight influenced by the threshold voltage variation in the CMOS transistor composing the starved inverter and current mirror structures, the overall variation in the oscillation frequency is primarily determined by the variations in memresistance of *M*_*i*_ if the supply voltage, *V*_DD_, is kept constant.

**Figure 5:**
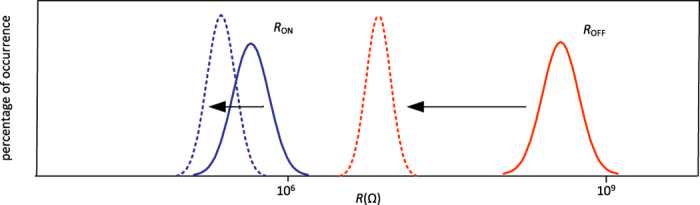
The effect of sneak path currents. The solid lines show resistance distribution of individual isolated memristors in ON and OFF state while the dashed lines show the readout resistance distribution after isolated memristors integrated in the crossbar array. The sneak path current will reduce the mean value of *R*_ON_ slightly and *R*_OFF_ significantly resulting in narrowed difference between ON and OFF resistance. Besides that, the sneak path current also reduces the standard deviation of resistance distribution in ON and OFF states.

**Figure 6:**
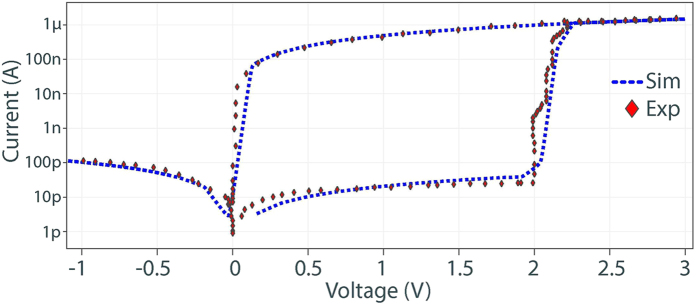
Current-voltage (*I*-*V*) characteristic for a memristor. The curves show the *I*-*V* relationship of a memristor with intrinsic diode characteristics. The red dotted line is obtained from experimental data^19^,^4^4 and the dashed line depicts the simulated results produced by our memristor model written in Verilog-A language and used in simulations in our study. For the SET operation (Fig. 1c) a positive voltage higher than 2 V (positive threshold voltage) is applied. For the RESET operation (Fig. 1c) a negative voltage lower than −3.5 V (negative threshold voltage) is applied.

**Figure 7:**
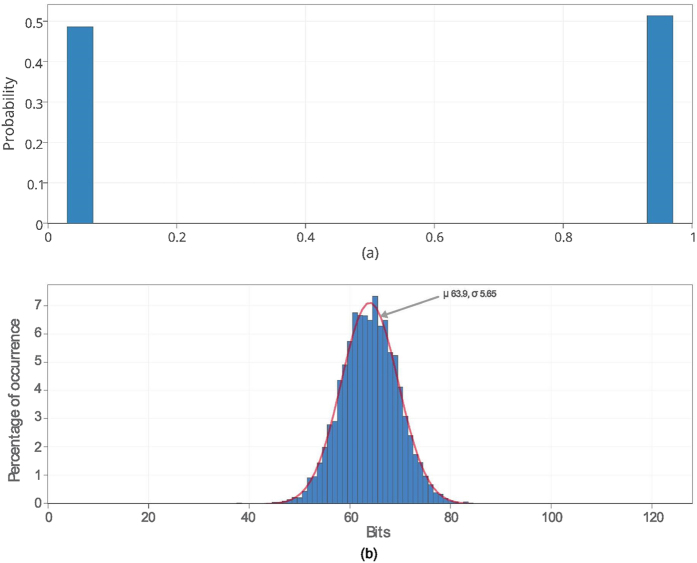
Uniformity and diffuseness. (**a**) Uniformity or randomness of mrSPUF: probability of output logic ‘1’ and ‘0’ are near 50% (50.76% and 49.24% for logic ‘1’ and logic ‘0’ respectively). (**b**) Hamming distance distribution for evaluating diffuseness: mean of HD is 63.9 bits out of 128 bits (49.96%). Standard deviation is 5.65 bits (3.63%).

**Figure 8:**
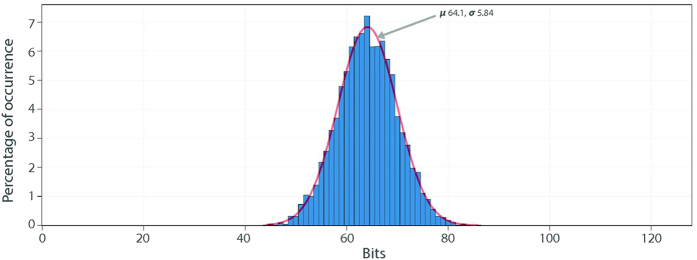
Uniqueness. Hamming distance distribution for evaluating uniqueness: mean of HD for intra-HD is 64.1 bits out of 128 bits (50.07%). Standard deviation is 5.84 bits (4.56%).

**Figure 9:**
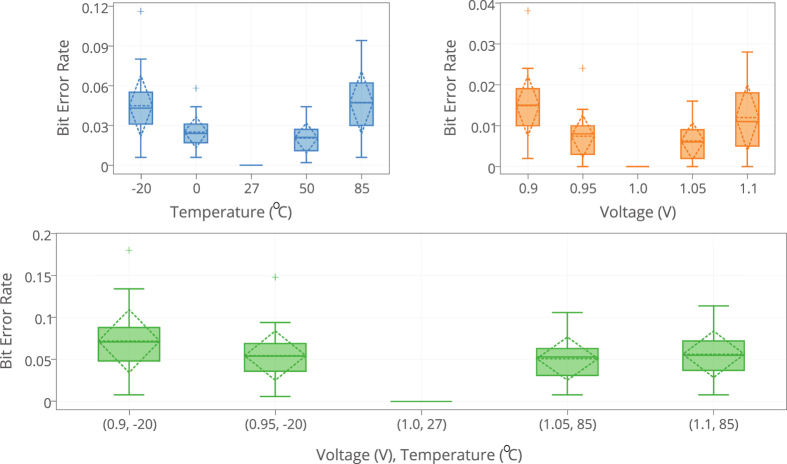
Bit error rate. BER of mrSPUF under different voltage and temperature deviations.

**Figure 10:**
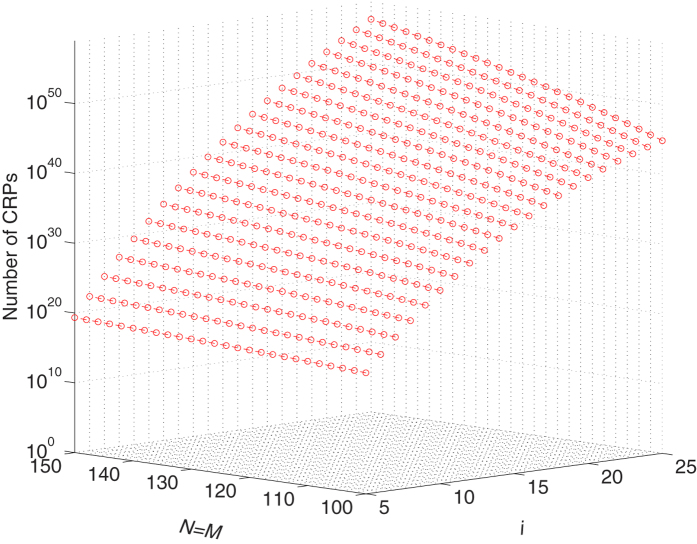
Number of CRPs. The number of CRPs vs the number of selected memristors, *i* and the array size *M*, *N* (*M* = *N*). As *i* and array size *M*, *N* increase, the number of CRPs increase almost exponentially.

**Figure 11:**
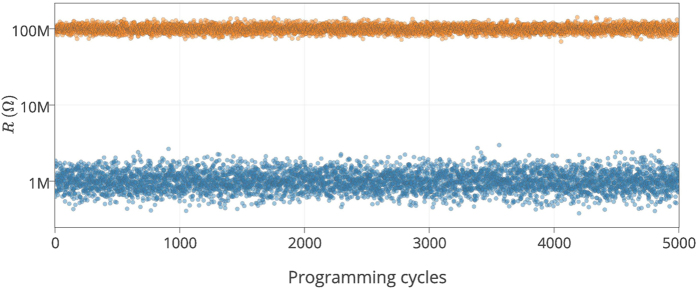
Cycle to Cycle (C2C) variations. *R*_OFF_ and *R*_ON_ variation of an individual memristor for 5000 cycles, experimental data is adopted from^24^.

We conduct extensive simulations based on experimental data to evaluate our proposed PUF architecture. The simulation study was carried out at the device level using Cadence tools. In these simulations the mrSPUF was built using a 40 × 40 nanocrossbar array with *r*_*w*_ = 1.25 Ω segment resistance for nano-wires and two 5-stage ROs as shown in Fig. 4. Each nano device is a memristor that is programmed to *R*_ON_ where the value of *R*_ON_ is randomly selected from a log-normal distribution, shown in Fig. 3 via Monte Carlo command in Cadence. It should be noted here that the log-normal distribution values are from extracted fabricated experimental data in^19^. Readout is achieved using a 1 V supply voltage to ensure the device memristance does not alter with respect to time as a result of the operation is below memristor’s threshold voltage. In these simulations, we use a standard CMOS 90 nm technology with a 1.0 V supply voltage. The memristor model is adapted from^40^,^4^1 written by Verilog-A language. The simulated results of memristor model with a large rectification ratio for minimizing sneak path currents shown in Fig. 6 agrees well with the experimental results.

### Fundamental PUF performance metrics

To assess the fundamental PUF performance of mrSPUF, metrics consist of uniformity, diffuseness, uniqueness and reliability are evaluated. More detailed definitions of these metrics can be found in^42^,^4^3.

∂ **Uniformity.** Uniformity is an indicator of the balance of ‘0’ and ‘1’ in the response vector. An ideal PUF should show that a ‘0’ or ‘1’ response is equiprobable. The uniformity is defined as:


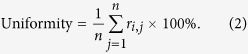


Where *r*_*i*,*j*_ is the *j*-th binary bit of an *n* bit response from a response vector *i*.

∂ **Diffuseness.** Diffuseness measures the difference between response vectors for different challenges applied to the same PUF. Diffuseness is measured by calculating the mean of HD for all the possible response vectors generated by the PUF. In reality, a random sample of response vectors are evaluated if the number of CRPs is too large. Diffuseness of ideal value is half of response vector length, ideally 50% in percentage. Diffuseness quantifies the information content that can be extracted from a PUF, in another words, how many IDs that this PUF has. If one PUF takes *m* IDs to evaluate its diffuseness, *R*_*i*_ and *R*_*j*_ (*i* ≠ *j*), have *n* bit responses, are two different response vectors corresponding to 2 different IDs. Then diffuseness is defined as:


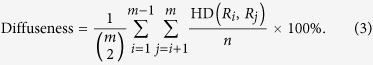


∂ **Uniqueness.** When applying the same challenge to different PUFs, the response vectors from different PUFs are expected to be different due to intrinsic variations of each PUF. This is a highly desirable characteristic that measures the amount of unique information that can be extracted from a PUF. Uniqueness is measured by inter-HD. If two PUFs, *i* and *j* (*i* ≠ *j*), have n bit responses, produce *R*_*i*_ and *R*_*j*_ response vectors corresponding to the same challenge, The uniqueness of *k* PUFs is defined as:


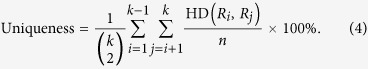


∂ **Reliability.** Reliability or steadiness indicates stability of PUF output bits, i.e. the ability to consistently generate the same response to a corresponding challenge. Reliability of an ideal value should be strong (100%) without. However, because noise (environmental variations, instabilities in circuit, aging) are unavoidable, there are always uncertain factors affecting the response.

A reference response Ref_*i*_ is recorded at normal operating condition (27 °C and 1.0 V supply voltage for our simulation), then a response vector 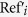 is extracted at a different operating condition but using the same challenge as before. After samples of 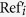 are collected, the HD between Ref_*i*_ and 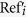 is calculated named intra-HD. The ideal value of intra-HD should be 0. Intra-HD can also be described by Bit Error Rate (BER), which is the percentage of flipped (error) bits out of response bits due to noise. If the same *n*-bit response is collected at a different operating condition with a value 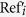. Note that *m* samples of 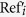 are collected. Then the average intra-chip HD or BER is defined as:


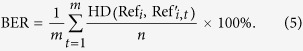


Where 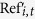 is the *t*-th sample of 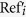. The reliability of a PUF is defined as:


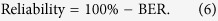


## Additional Information

**How to cite this article**: Gao, Y. *et al.* Memristive crypto primitive for building highly secure physical unclonable functions. *Sci. Rep.* **5**, 12785; doi: 10.1038/srep12785 (2015).

## Figures and Tables

**Figure 2:**
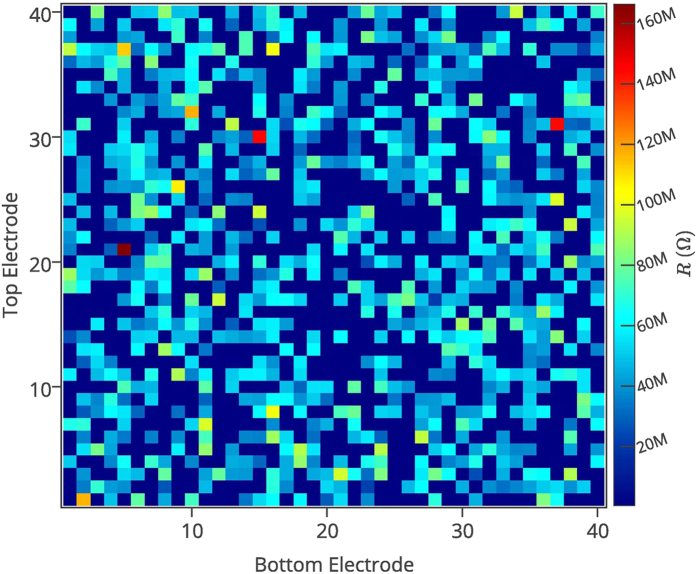
Resistance variation in a nanocrossbar. Programming variation across a 40 × 40 nanocrossbar. Each crosspoint is a memristor that can be accessed and programmed using a top electrode and a bottom electrode. Memristors are randomly programmed into *R*_ON_ and *R*_OFF_ state. Experimental data is obtained from^19^.

**Table 1:** Comparison mrSPUF with other memristor based PUFs.

	34	35	mrSPUF^45^,^46^,^47^
Uniqueness	≈50%	≈50%	50.07%
Uniformity	N/A	≈50%	50.76%
Number of CRPs	*M* × *N*	*M*	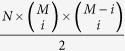
Nanocrossbar used	✓	x	✓
Security analysis	x	x	✓
Strong PUF candidate	x	x	✓
Reconfigurable PUF	x	x	✓
